# Jacalin Bound Plasma O-Glycoproteome and Reduced Sialylation of Alpha 2-HS Glycoprotein (A2HSG) in Rheumatoid Arthritis Patients

**DOI:** 10.1371/journal.pone.0046374

**Published:** 2012-10-03

**Authors:** Ashish Saroha, Saravanan Kumar, Bishnu P. Chatterjee, Hasi R. Das

**Affiliations:** 1 Genomics and Molecular Medicine Division, CSIR- Institute of Genomics and Integrative Biology, Delhi, India; 2 Department of Natural Science, West Bengal University of Technology, Kolkata, West Bengal, India; Drexel University College of Medicine, United States of America

## Abstract

Glycosylation studies of plasma proteins can reveal information about the onset and progression of diseases, where in the glycan biosynthetic pathways are disturbed as in rheumatoid arthritis (RA). The present study was focused on analysis of O-linked glycoproteins of plasma in RA patients. Two dimensional gel electrophoresis of jacalin bound plasma of RA patients revealed a number of differentially expressed protein spots as compared to healthy controls. Eighteen protein spots were found to have statistically significant (*p*<0.05) difference in their expression level from four sets of gels and were identified by MALDI-TOF MS. Most of the identified proteins were predicted to be O-glycosylated proteins by Net–O-Gly 3.1 algorithm. Among these the alpha 2HS glycoprotein (A2HSG) was found to be down regulated whereas inter alpha trypsin inhibitor H4 (ITIH4) was up regulated and this was validated by Western blotting. The glycosylation studies showed the reduced N-linked sialylation of A2HSG in RA patients. Altered glycoprotein expression and functional as well as structural studies of glycans might help in the diagnosis of RA and understanding the disease pathogenesis.

## Introduction

Glycoproteins play important roles in cellular functions like cell to cell adhesion, cell migration, cell signaling, immunity, and various other intracellular processes through their glycans [1]–[3]. Cellular factors play a pivotal role in the regulation of the glycosylation reactions in cells. Alterations in the cellular environment affect the activity of various glycosidases and glycosyltransferases leading to the aberrant glycosylation [4]–[6]. Many subtle changes in functions of glycoproteins were found to be attributable to changes in composition or structure of the glycan moieties. Therefore, interest in glycoproteomic analysis has gained momentum to identify the glycan based disease markers. As plasma is an important reservoir of glycoproteins and reflects the patho-physiological condition of the body, the plasma glycoproteins hold a great potential to recognize disease specific glycan based molecular fingerprints [7], [8]. Currently, disease related biomarker discovery has grown dramatically to replace sub optimal *in-vitro* diagnostic assays.

Rheumatoid arthritis (RA) is an autoimmune, chronic, systemic, inflammatory rheumatic disease characterized by pain and swelling of synovial joints leading to their deformation and destruction [9]. The most notable changes during the development of RA are alterations of glycosylation in plasma proteins and their level of expression [10]. The increased level of agalactosylated IgG (IgG0) correlates with the disease severity in patients of RA [11], [12]. Micro heterogeneity in glycosylation of IgG and many other plasma proteins have been widely studied and are implicated in the pathogenesis of RA [13]–[18]. These changes in glycosylation activate the immune system and lead to chronic inflammation of the synovial membrane [19].

The present study was aimed at identifying glycomarkers by glycoprotein analysis of the plasma proteins of RA patients. The enrichment of O-linked glycoproteins of plasma was carried out by using jacalin affinity chromatography, followed by protein profiling by 2-DE. The jacalin binds to O-linked GalNAc alpha1-peptide where C6-OH of <alpha>-GalNAc is free and is not bound/or in a glycosidic bond to another sugar residue like Tn-antigen (GalNAc<alpha>1-ser/thr), core-1 (Gal<beta>1–3GalNAc<alpha>1-ser/thr or T-antigen), sialyl T (Neu5Ac<alpha>2–3GalNAc<alpha>1-ser/thr) and core-3 (GlcNAc<beta>1–3GalNAc<alpha>1-ser/thr) structures [20]. Jacalin doesn’t bind to all types of O-glycans (as mentioned above) and binds also to high mannose N-glycans. Although other lectins like *Helix pomatia* agglutinin (HPA) and peanut agglutinin (PNA) are also known to bind O-linked glycans [21], [22]. The jacalin has some advantages over these lectins such as high specificity and easy availability at comparatively low cost. Unlike PNA, jacalin binds sialylated glycans albeit poorly. The differentially expressed plasma proteins in RA patients and healthy controls were identified by MALDI-TOF MS. Additionally, using bioinformatic tools, functional categorization and prediction of O-glycosylation sites in differentially expressed proteins were evaluated. The expression levels of two inflammation related proteins such as, alpha 2HS glycoprotein (A2HSG) and inter alpha trypsin inhibitor H4 (ITIH4) were validated by Western blotting.

## Results

### O-linked Glycoproteins, 2-DE and MS Identification

**Figure 1:**
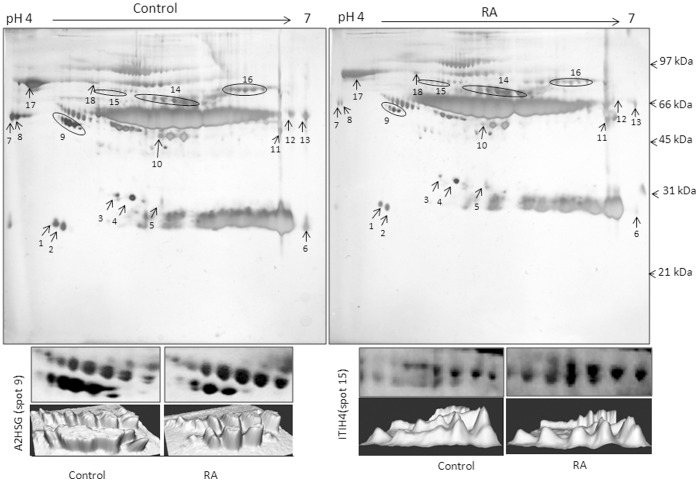
Two dimensional gel electrophoresis (2-DE) of jacalin bound plasma. A representative silver stained 2-DE gel image of jacalin agarose bound plasma proteins from four sets of pooled samples from controls and RA patients. The samples were pooled as described in materials and methods section. The gels were analyzed for differential expression of proteins using PD-Quest software and the differentially expressed proteins were labelled by arrows. The lower panel showed zoomed image and 3D view of A2HSG (spot no 9) and ITIH4 (spot no 15).

To enrich O-glycosylated plasma proteins, jacalin affinity column was used. The 2-DE analysis of jacalin bound plasma proteins of control and RA patients was carried out to identify the differentially expressed protein spots (Figure 1). The software analysis of 2-DE gels by PD-Quest showed many spots present in control and RA patients. The changes of protein expression in RA patients were calculated with respect to control from four sets of gels. Eighteen spots showing ≥1.5 fold difference (*p*<0.05) in the expression level, were considered and identified by MALDI-TOF MS (Table 1, Figure S1).

**Table 1:** List of differentially expressed proteins, as identified by MALDI-TOF MS, in plasma of RA patients with respect to normal control.

Spot No.	Protein identified	*M.W/ pI	Accession number	Peptides matched/ unmatched	Mowsescore	Sequence coverage (%)	Foldchange	Biologicalfunction
1	Immunoglobulin J chain	16,041/4.6	P01591	6/6	102	53	↓ 2.4	Antigen binding
2	Immunoglobulin J chain	16,041/4.6	P01591	8/15	131	62	↓ 1.7	Antigen binding
3	Alpha-1-microglobulin	39,886/5.9	P02760	9/10	92	25	↓ 2.9	IgA and heme binding
4	Alpha-1-microglobulin	39,886/5.9	P02760	11/22	95	28	↓ 1.6	IgA and heme binding
5	Sequence 278 from Patent WO0222660	34,016/5.1	NA	13/4	191	59	↓ 2.0	RNA processing
6	Immunoglobulin kappa chain C region	11,608/5.5	P01834	10/12	157	66	↓ 20.0	Complement activation
7	Immunoglobulin alpha-1- chain C region	54,327/8.0	P01876	21/9	271	52	↓ 7.1	Antigen binding
8	Immunoglobulin alpha-1- chain C region	54,327/8.0	P01876	22/21	249	59	↓ 6.6	Antigen binding
9	Alpha-2-HS-glycoprotein precursor	40,098/5.4	P02765	10/3	143	32	↓ 2.2	Acute phase response
10	Fibrinogen gamma chain precursor	52,106/5.3	P02679	14/47	129	44	↓ 3.8	Protein polymerization
11	CLL-associated antigen KW-11	53,557/4.9	Q8IWJ2	38/9	501	74	↑ 1.5	Microtubule organising
12	Immunoglobulin alpha-1- chain C region	54,327/8.0	P01876	13/4	186	35	↓ 6.6	Antigen binding
13	Immunoglobulin alpha-1- chain C region	54,327/6.7	P01876	10/15	112	37	↓ 3.1	Antigen binding
14	Hemopexin precursor	52,385/6.5	P02790	11/12	123	29	↑ 1.6	Iron homeostasis
15	Inter-alpha-trypsin inhibitor heavy chain H4 precursor(ITI heavy chain H4)	103,522/6.5	Q14624	11/33	87	19	↑ 1.8	Acute phase response
16	Immunoglobulin mu chain C region	50,210/6.3	P01871	13/26	105	22	↓ 2.2	Antigen binding
17	Plasma protease C1 inhibitor	56,980/5.9	P05155	13/7	156	22	↑ 1.5	Complement activation
18	Complement C1s	78,174/4.8	P09871	13/7	142	20	↓ 1.9	Complement activation

* M.W and p*I* were estimated on the basis of the sequence submitted in swiss-prot database.

↑ indicates up regulation and ↓ indicates down regulation of proteins in RA with respect to control.

### Validation of Differentially Expressed Proteins by Western Blotting

**Figure 2:**
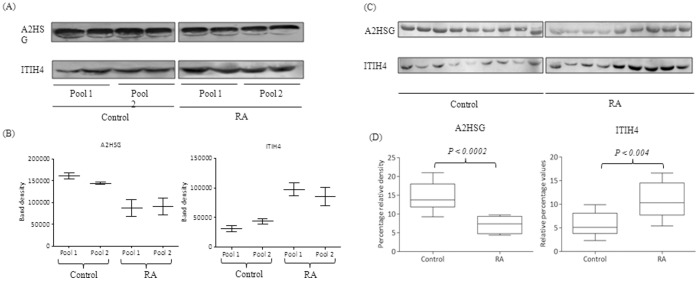
Western blot analysis of A2HSG and ITIH4. (A) In RA (n = 35), pool 1 and pool 2 of group 1 consisted of 7 and 8 samples respectively while each pool of group 2 contained 10 samples. Similarly, in control (n = 30) each pool of group 1 and group 2 consisted of 10 and 5 samples respectively.(B) Mean density of each protein band in the Western blot followed by statistical analysis using One way ANOVA (*p*<0.05) showed the change in the expression of protein. (C) Representative Western blot image of A2HSG and ITIH4 from individual Control and RA samples. (D) Mean percentage relative density of each protein band in control and RA were plotted on Y-axis. Statistical analysis was done using Mann-Whitney U test (*p*<0.05).

Of 18 identified proteins, one up regulated ITIH4 and one down regulated protein A2HSG in plasma of RA patients, were selected for further validation by immunobloting as these are known to be associated with inflammation [23], [24]. Since the enrichment of plasma glycoproteins by jacalin chromatography may not reflect the actual difference in the expression of the protein, the expression levels of A2HSG and ITIH4 were checked in plasma without jacalin enrichment by Western blotting (Figure 2A, C). The densitometric analysis of Western blots showed that the changes in the levels of expression were statistically significant (Figure 2B, D). However, the fold differences before (Western blotting) and after (2-DE) jacalin binding varies for both the proteins.

### Bioinformatics Analysis

The O-glycosylation site prediction analysis using Net-O-Glyc algorithm of the jacalin bound glycoproteins, as detected in the gel, revealed that most of the identified proteins had O-glycosylation sites (Table 2). The Net-O-glyc server predicts only mucin type GalNAc O-glycosylation sites in mammalian proteins. However, there are other O-glycans present in proteins as well [25]. The functional categorization of these proteins showed most of them to be of immunological importance (Table 1).

**Table 2:** Prediction of O-glycosylation sites using Net-O-Glyc3.1 program.

S. No.	Proteins	Predicted O-glycosylation sites
1	Immunoglobulin J chain	T-127,T-130
2	Alpha-1-microglobulin	T-24
3	Immunoglobulin kappa chain C region	T-1,S-6
4	Immunoglobulin alpha-1-chain C region	S-2,T-4,S-5,T-98,S-105,T-106,T-109,S-111,S-113,T-114,T-117, S-119,S-121,T-161,T-163,T-201,T-203,T-205,T-213,T-216,T-225,
5	Alpha-2-HS-glycoprotein precursor	T-256,S-257,T-270,S-280,S-293,T-339,T-341
6	Plasma protease C1 inhibitor	T-71,T-72,T-76,T-79,T-82,T-83,T-87,T-88,T-91,T-92,T-95,T-96,T-99,T-103,T-106,T-107,T-111,S-113,T-115,T-118,T-119,T-128
7	Cutaneous T-cell lymphoma-associated antigen 5	T-7,T-687,S-731,S-780,S-784,T-795,T-804
8	Hemopexin precursor	T-24,T-40
9	Immunoglobulin mu chain C region	S-4,T-7,T-379
10	Inter-alpha-trypsin inhibitor heavy chain H4 precursor(ITI heavy chain H4)	T-701,T-722
11	Complement C1s	T-544

### Altered Glycosylation of A2HSG

**Figure 3:**
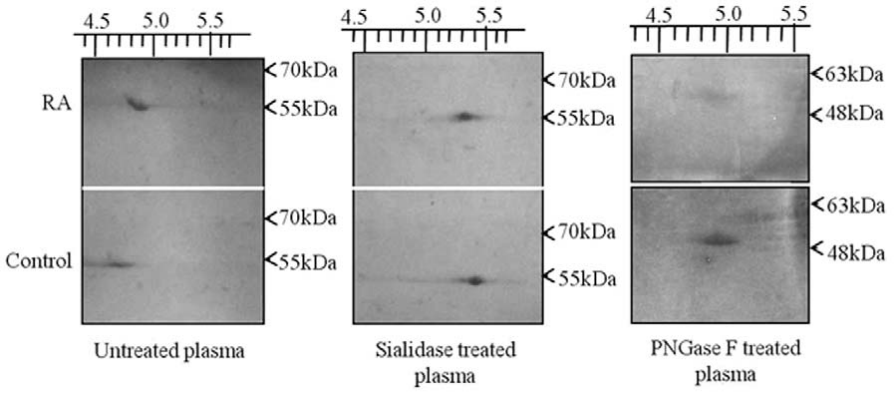
Glycosylation studies in A2HSG. 2-DE Western blot of A2HSG in untreated plasma showed the higher p*I* of A2HSG in RA patients compared to control. The treatment with sialidase shifted the p*I* of control towards higher p*I* in comparison to that of RA, while the shift in p*I* was found to be equal in both the samples after PNGase F treatment. The experiment was repeated thrice with different pooled samples.

**Figure 4:**
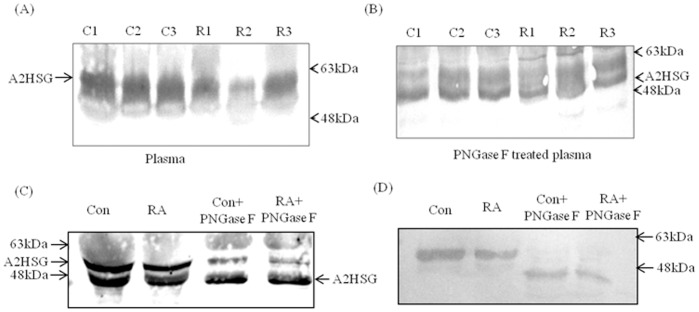
Glycosylation studies in A2HSG by lectin blotting. The binding of sialic acid specific lectin (MAA, *Maackia amurensis*) to A2HSG in (A) untreated (B) PNGase F treated plasma. Same pooled samples were used for lectin blot (C) and Western blot using anti-A2HSG antibody (D) to locate the protein before and after enzyme treatment. C1, C2 are pool 1 and 2 of control group 1 respectively while C3 represents pool 1 of control group 2. R1, R2 are pool 1 and 2 of patient group 1 respectively while R3 represents pool 1 of patient group 2.

Sialic acid is an important charged sugar present on both N- and O-linked glycan structures in A2HSG. To study the sialylation changes in A2HSG, we used a strategy involving 2-DE immunoblotting of plasma (without jacalin enrichment) after treatment with glycosidases. Immunoblotting after 2-DE showed the difference in isoelectric points (p*I*) of A2HSG in control and RA patients. The p*I* was manually calculated by equally dividing the entire length of the pH strip (4–7) containing the gel. The p*I*s of A2HSG in control and RA patients were found to be 4.5 and 4.7 respectively (Figure 3). Sialidase treatment resulted in shift of p*I* of A2HSG in both control and RA patients to 5.3 each. This change in p*I* appeared to be due to decrease in the level of sialic acid. PNGase F digestion of plasma was carried out to ascertain whether the decrease in the level of sialic acid is associated with N- or O-linked glycan. The PNGase F digestion shifted the p*I*s to 4.8 in both controls and RA patients suggesting the reduced sialylation of A2HSG in N-linked glycans in RA. Sialylation status of A2HSG by MAA (*Macckia ammurensis* agglutinin, specific to Neu5Ac α2, 3 glycans) blotting indicated significant reduction in binding of MAA with A2HSG in patients’ plasma (Figure 4A). Lectin (MAA) blotting of plasma after PNGase F treatment also showed better binding with control compared to RA patients. However, the difference in MAA binding between control and patients’ plasma reduced significantly after enzyme treatment. These results indicated the 0.3 fold sialylation (Neu5Ac α2, 3) difference in N-linked glycans of A2HSG in RA patients (Figure 4B).

## Discussion

Profiling of plasma proteins in RA can be very valuable in understanding disease pathology and can provide potential marker for disease. In this report, the comprehensive comparative analysis of jacalin bound mucin type O-linked plasma glycoproteins in RA patients and normal controls was done using advanced proteomic tools like 2-DE and MS. O-linked glycosylation has been widely studied in various cellular processes and diseases [26], [27]. To the best of our knowledge, this is the first study showing the comparative profiling of jacalin bound O-linked plasma proteins in RA patients. Jacalin has been used for the enrichment of plasma IgA1, which has many O-linked glycosylation sites [28], and was observed as thick blotch in 2-DE. Besides IgA1, many differentially expressed proteins were found in jacalin bound plasma of RA patients as compared to healthy controls. The identification of these differentially expressed proteins by MS revealed most proteins to be of immunological importance. Lectin affinity chromatography sometimes may result in non specific binding [29]. Moreover, no consensus recognition sequence for O-glycosyltransferases is known; therefore, analysis of jacalin bound proteins was carried out using NetOGlyc 3.1 to ensure the presence of O-linked glycosylation sites. The *in silico* analysis revealed that most of the jacalin bound proteins possessed potential O-glycosylation sites. Since A2HSG and ITIH4 have been reported to be associated with inflammation [23], [24], we selected their immune reactivity and expression level in the plasma of RA patients by Western blotting. Both the proteins indeed were found to be immunologically reactive indicating them to be important in RA.

ITIH4 is a newly discovered member of inter alpha trypsin inhibitor family of heavy chains and is also called as plasma kallikrein-sensitive glycoprotein (PK120) or inter-alpha-trypsin inhibitor family heavy chain-related protein [30]. It has two potential O-linked glycosylation sites. Unlike other heavy chain members of ITI family (ITIH1-7), ITIH4 exists as a free heavy chain isoform in blood circulation [31]. It is a 120 kDa protein which is cleaved into 85 kDa N-terminal and 35 kDa C-terminal fragment by plasma kallikarein system. The 85 kDa fragment is further cleaved into 57 kDa and 28 kDa fragments. The above proteolytic processing of ITIH4 varies in some cancers [32]. Mohamed *et al* studied the level of 35 kDa fragment in various types of carcinomas [33]. We observed that the level of 85 kDa fragment was found to be increased by two fold in plasma of RA patients. The *in vivo* function of ITIH4 is not known but the structural analysis revealed that it contains potential actin and calcium binding sites suggesting its role in inhibition of actin polymerization and calcium metabolism. Additionally, this protein was also found to block the phagocytosis of polymorphonuclear blood cells indicating its anti-inflammatory role [34], [35]. Significantly elevated level of ITIH4 in the sera of postmenopausal women was shown to predict its role in calcium metabolism and prognostic value as a marker for increased osteoclast activity and bone fracture [36].

A2HSG, a human homologue of bovine fetuin-A, is synthesized in liver but enriched in bones suggesting its role in regulation of osteogenesis [37]. A2HSG acts as an inhibitor of ectopic calcification [38]. It plays a role in augmentation of phagocytosis of neutrophils by macrophages, thus acting as an anti-inflammatory molecule [39], [40]. In our study, the level of plasma A2HSG was decreased by two fold in RA patients as compared to normal controls. The earlier reports have shown a 75% reduction in the level of A2HSG in hepatocytes in the presence of pro-inflammatory cytokine IL-6. A probable explanation for the decreased level of serum A2HSG in RA patients may be due to high level of IL-6 [41]. The reduced level of A2HSG has also been reported to be associated with the vascular calcification and consequently the increased risk of cardiovascular diseases [42].

A2HSG contains both N- and O-linked glycan sites. Due to the lack of specific enzyme to separate the intact O-linked glycans, an alternate strategy was used to study site specific (N/O linked) sialylation. The treatment with PNGase F and *Arthrobacter ureafaciens* sialidase and 2-DE revealed the reduced sialylation of N-glycans of A2HSG in plasma of RA patients. Sialylation plays an important role in structural and functional establishment of synaptic pathways [43], leukocytes rolling and extravasation during inflammation [44]. The reduced sialylation activity was shown in various clinical conditions like metastasis of cancer cells [45], IgA1 nephropathy [46], and pancreatic cancer [47]. In the case of secretory proteins, like A2HSG, the desialylation accelerates the process of degradation of proteins by interaction with asialoglycoprotein receptors present on the hepatic cell membrane [48]. Various other pro-inflammatory cytokines including IL-6 released in RA are known to affect the glycosylation of several acute-phase proteins by a mechanism independent of that regulating their rate of synthesis [49]. Here, the reduction in sialylation may not be limited to A2HSG only but may be occurring in other proteins as well. Przybysz *et al* reported an altered sialylation and fucosylation of synovial and plasma fibronection in RA patients [50]. Desialylation of A2HSG may have a major effect on the protein structure and its functions, such as bone calcification, ossification and cell adhesion as the interaction of A2HSG with other molecules is mediated through sialylation [51].

## Materials and Methods

### Ethics Statement

All the participants involved in the study gave written informed consent and the human ethics committees of Research and Referral Army hospital and Institute of Genomics and Integrative Biology approved this study.

### Sample Collection

Blood samples from 35 RA patients fulfilling the revised criteria of American College of Rheumatology [52] for the disease, were collected from the Department of Rheumatology, Research and Referral Army Hospital, New Delhi, India. All the patients gave informed consent and the human ethics committee from hospital and the institute approved this study. Blood samples from 30 healthy individuals served as controls. The age in the control and RA patient groups ranged from 30–45 and 30–50 years respectively. In case of RA, samples were divided into two groups considering the age 30–40 years (15 samples, group 1) and 41–50 years (20 samples, group 2) as shown in Table S1. Similarly, the controls were also divided in two age groups, 30–40 (20 samples, group 1) and 41–45 (10 samples, group 2). Each group was further divided in two pools. In RA, pool 1 and pool 2 of group 1 consisted of 7 and 8 samples respectively while each pool of group 2 contained 10 samples. Similarly, in control each pool of group 1 and group 2 consisted of 10 and 5 samples respectively. The Western blot analysis of individual samples was also done. The plasma was separated from the blood after centrifugation at 2500×g for 15 min at 4°C. Plasma samples were aliquoted and stored at −80°C till further use.

### Enrichment of Plasma Glycoproteins

Prior to glycoprotein enrichment, 500 µl of plasma sample from each pool of RA patients and healthy controls was diluted with 10 mM TBS (pH 7.2) and loaded separately on spin columns containing 500 µl of jacalin agarose beads (Vector laboratories, USA). After incubating for 30 min at room temperature, unbound proteins were removed by spinning the column. The column was washed with TBS till the OD at 280 nm reached to zero and the bound proteins were eluted by TBS buffer containing 0.8 M galactose and 500 µl of each fraction was collected. All the fractions were pooled and buffer exchanged against TBS (10 times diluted) and concentrated using 5 kDa protein concentrator (Amicon Inc, USA). The concentration of jacalin bound protein was estimated using Bradford protein assay [53].

### Two Dimensional Gel Electrophoresis (2-DE), Protein Visualization and Image Analysis of Gels

Three hundred microgram of jacalin bound plasma from each group of controls and RA patients were resuspended in 300 µl of rehydration buffer (8 M urea, 4% CHAPS, 65 mM DTT, and 0.25% ampholytes). The samples were rehydrated on 17 cm IPG strip, pH 4–7 overnight followed by IEF (Protean IEF cell, Bio-Rad, USA) at 10,000 V till 50,000 Vh were reached. The strips were then equilibrated for 20 min in reduction buffer (6 M urea, 1.5 mM Tris-HCl (pH 8.8), 2% (w/v) SDS, 2% (w/v) DTT and 20% (w/v) glycerol) followed by replacement with alkylation buffer (6 M urea, 1.5 mM Tris-HCl (pH 8.8), 2% (w/v) SDS, 2.5% (w/v) IAA and 20% (w/v) glycerol) for 20 min. The second dimension separation was carried out in 12% polyacrylamide gel using Protean IIxi multi-cell apparatus (Bio-Rad, USA) at a constant current of 50 mA per gel till the tracking dye reached near bottom age of the gel. Mass spectrometry compatible silver staining of gels was performed and scanned as 16 bit grey scale images (Alpha Digi doc 1201 scanner, Alpha Innotech Corp, USA) [13]. 2D gels of both control and RA patients were run and silver stained in parallel to avoid any variation. Intensity of each protein spot was manually determined using alpha digi doc software and the normalization of the spot intensities was done by the total optical density in the gel *i.e.* the normalized intensity was the percentage of the intensity of each spot over the sum of intensities of all detected spots in the gel. The fold change in expression of each statistically matching spot in RA with respect to control was calculated.

### In-gel Digestion and Protein Identification by Matrix Assisted Laser Desorption Ionization Time of Flight Mass Spectrometry (MALDI-TOF MS)

Silver stained protein spots from 2-DE gels were excised, cut into small pieces and processed for in-gel digestion as described earlier [13]. Briefly, gels pieces were washed in destaining solution (0.2% K_3_FeCN_6_ and 0.04% Na_2_S_2_O_3_) till the brown colour disappeared completely, followed by washing with 100 mM NH_4_HCO_3_ and 100% CH_3_CN in 1∶1 ratio. Finally, gel pieces were dehydrated in 100% CH_3_CN and dried completely in speed vac (Thermo savant, USA) for 20 min. Ten microlitre of sequencing grade trypsin (20 ng/µl), prepared in 25 mM NH_4_HCO_3,_ was added to gel pieces and kept at 37°C for 16 h for digestion of proteins. The supernatant containing peptides was collected and transferred to the fresh microfuge tubes rinsed with CH_3_CN. Equal volumes (10 µl) of 0.1% TFA and CH_3_CN were added and sonicated for 15 min at room temperature. Supernatant was collected and dried completely in speed vac and stored at −80°C till MS analysis. For MS analysis the peptides were resuspended in 10 µl of 0.1% TFA containing 30% CH_3_CN. One microlitre of peptide solution was mixed with an equal amount of α-cyano hydroxy cinnamic acid (CHCA) matrix solution prepared in 70% CH_3_CN and 30% 0.1%TFA. One microlitre of the mixture was spotted on MALDI ground steel target plate and mass spectrum was obtained on Ultraflex II MALDI-TOF/TOF mass spectrometer equipped with a pulsed N_2_ laser (337 nm). Operating conditions were as follows: ion source 1 = 19.00 kV, ion source 2 = 16.50 kV, lens voltage = 20.00 kV, optimized pulsed ion extraction time = 120 ns, matrix suppression = 400 Da and positive reflectron mode. Around 500 laser shots were collected from one spot from five different positions with 100 shots per position. Spectra were externally calibrated with the following peptide masses - angiotensin II (M+H)^ +^mono = 1046.54180, angiotensin I (M+H)^+^ mono = 1296.684 80, bombesin (M+H)^+^mono = 1347.73450, ACTH Clip (1–17) (M+H)^+^ mono = 2093.08620, somatostatin (28)(M+H)^+^mono = 3147.47100. Peptide mass fingerprinting and MS/MS (fragmentation) spectra were searched online against NCBInr, Swiss-prot and mascot (MSDB) databases using the mascot search engine (Matrix Science, London) with following parameters: mass tolerance for PMF = 100 ppm, mass tolerance for MS/MS = 0.5 Da, fixed modification = carbamidomethylation, variable modification = methionine oxidation and missed cleavage = 0–1.

### Western Blot Analysis

Equal amount of proteins (50 µg) from plasma of controls and RA patients were separated by sodium dodecyl sulphate polyacrylamide gel electrophoresis (SDS-PAGE). After the separation, proteins were electro blotted onto nitrocellulose membrane using a semi-dry apparatus (Bio-Rad, USA). The membranes were blocked with 5% BSA (w/v) prepared in phosphate buffered saline containing 0.1% Tween -20 (PBST) for 1 h at room temperature, washed and were separately incubated with the following antibodies: anti-human alpha 2HS glycoprotein (Acris antibodies, GmbH) and anti human ITIH4 (Santa Cruz Biotechnology) each diluted to 1∶5000 using blocking buffer. After washing with PBST the membrane was incubated with HRP conjugated anti-mouse/anti-goat IgG secondary antibody (dilution 1∶10,000) prepared in PBST. Reactive protein bands were visualized by exposing the membrane to X-ray film (Fuji films, Japan) for 15, 30 and 60 sec using ECL Western blotting detection kit (Santa Cruz biotechnology). Western blotting of each protein was performed in duplicates. For individual samples the percentage relative density of A2HSG and ITIH4 was calculated by normalising with the density of total proteins of each sample as appeared in the ponceau staining (Figure S2, Table S2).

### Bio-informatics Analysis

The O-glycosylation sites in the proteins were predicted by using Net-O-glyc 3.1 algorithm (http://www.cbs.dtu.dk/services/NetOGlyc). The Net-O-glyc server produces neural network predictions of only mucin type GalNAc O-glycosylation sites in mammalian proteins [54].

### Glycosidases Treatment of Plasma Proteins

Fifty microgram of plasma proteins of control and RA patients were separately digested with PNGase F from *Flavobacterium meningosepticum* (Sigma chemicals) in a digestion buffer containing 1% Triton X-100 and 10 mM β-mercaptoethanol. The protein solution was heated at 100°C for 10 min and cooled before adding 5 µl (0.5U µl) of PNGase F (Sigma chemicals). Digestion was carried out at 37°C for 3 h. Reaction was stopped by heating the solution at 100°C for 5 min. The digestion of plasma proteins (50 µg) was carried out using 3 µl of sialidase from *Arthrobacter ureafaciens* (Sigma chemicals) in 50 mM sodium phosphate buffer pH 5.8 for 3 h. The reaction was stopped by heating at 100°C for 5 min.

### Lectin Blotting

After transferring the proteins from SDS-PAGE gel to the NC membrane, the membrane was blocked with 3% BSA prepared in PBST. After washing the membrane was treated with 1 µg/ml of biotinylated *Macckia ammurensis* agglutinin (MAA) prepared in PBST at room temperature for 1 h. The membrane was washed again 3 times with PBST and streptavidin peroxidase (1 µg/ml) was added and kept for 1 h at room temperature. The blots were developed using developing solution, which contained 4-chloro napthol (1 mg/ml) and 0.1% of H_2_O_2_ prepared in 20% methanol in PBS.

### Statistical Analysis

Protein spots showing change in the expression (≥1.5 fold) between two groups at the significance level of *p*<0.05 were considered as statistically significant. Results were expressed as mean ± S.D. in the Western blots. Statistical analysis was performed by student’s *t-*test and one way ANOVA. For individual samples the statistical analysis was made by Mann whitney U test where *p<0.05* was considered as statistically significant. All the statistical analysis was done using Graphpad prism 5.0 (Graphpad software).

### Conclusion and Future Directions

Glycans hold immense potential for disease diagnosis as the changes in the physiological condition of the cell affect the protein glycosylation machinery. Studies on glycobiomarkers converge with research on how distinct carbohydrates determinants are turned into bioactive signals. The O-glycoproteomic strategy, involving jacalin enrichment and digestion with various glycosidases, was found to be very useful in investigating the altered expression and glycosylation of plasma proteins in RA. The functional and structural characterization of the altered glycans will help in understanding the role of protein glycosylation in disease which may pave the way for novel therapeutic targets.

## Supporting Information

Figure S1 **MALDI-TOF MS analysis of alpha 2-HS glycoprotein (A2HSG).** (A) PMF spectrum (B) Online database search result of PMF spectrum showing the significance of the result (C) masses of peptides matching to the protein and (D) complete sequence of the protein and position of the peptides matching to the protein are underlined.
(TIF)

Figure S2 **Ponceau image for densitometric analysis.** Ponceau stained image of individual controls and RA patients after transferring the proteins to nitrocellulose membrane.
(TIF)

Table S1 **Demographic data of RA patients.**
(DOC)

Table S2 **Densitometric analysis of images.** Densitometric data of ponceau stained and anti-A2HSG and anti-ITIH4 blot images of individual controls and RA patients as calculated by alpha digidoc algorithm.
(XLS)
