# Supplementary figures and images for: Jacalin Bound Plasma O-Glycoproteome and Reduced Sialylation of Alpha 2-HS Glycoprotein (A2HSG) in Rheumatoid Arthritis Patients
Source: PLoS One. 2012 Oct 3;7(10):e46374. doi: 10.1371/journal.pone.0046374 (PMC3463590; doi:10.1371/journal.pone.0046374)

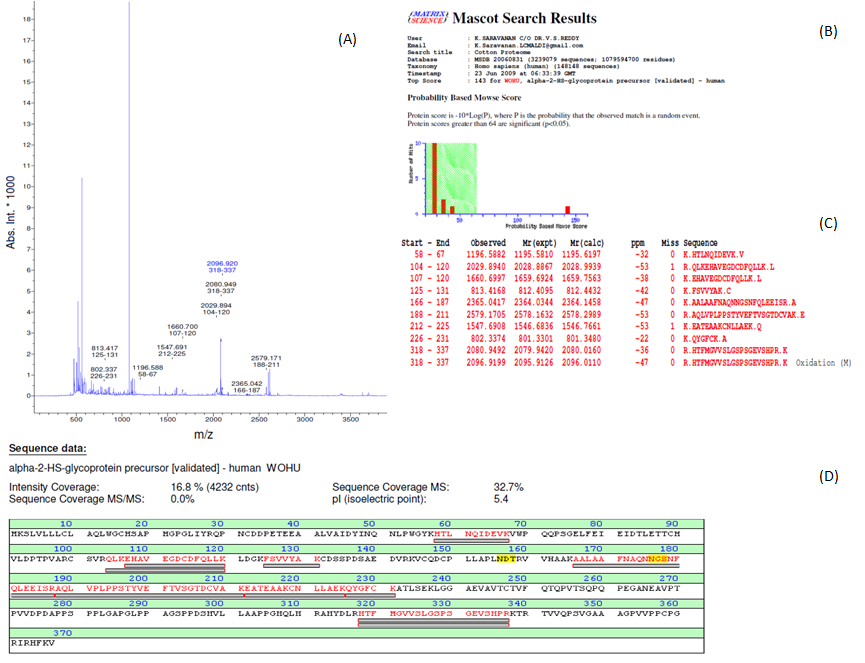

Supplement: Figure S1 — MALDI-TOF MS analysis of alpha 2-HS glycoprotein (A2HSG). (A) PMF spectrum (B) Online database search result of PMF spectrum showing the significance of the result (C) masses of peptides matching to the protein and (D) complete sequence of the protein and position of the peptides matching to the protein are underlined. (TIF) [file pone.0046374.s001.tif]

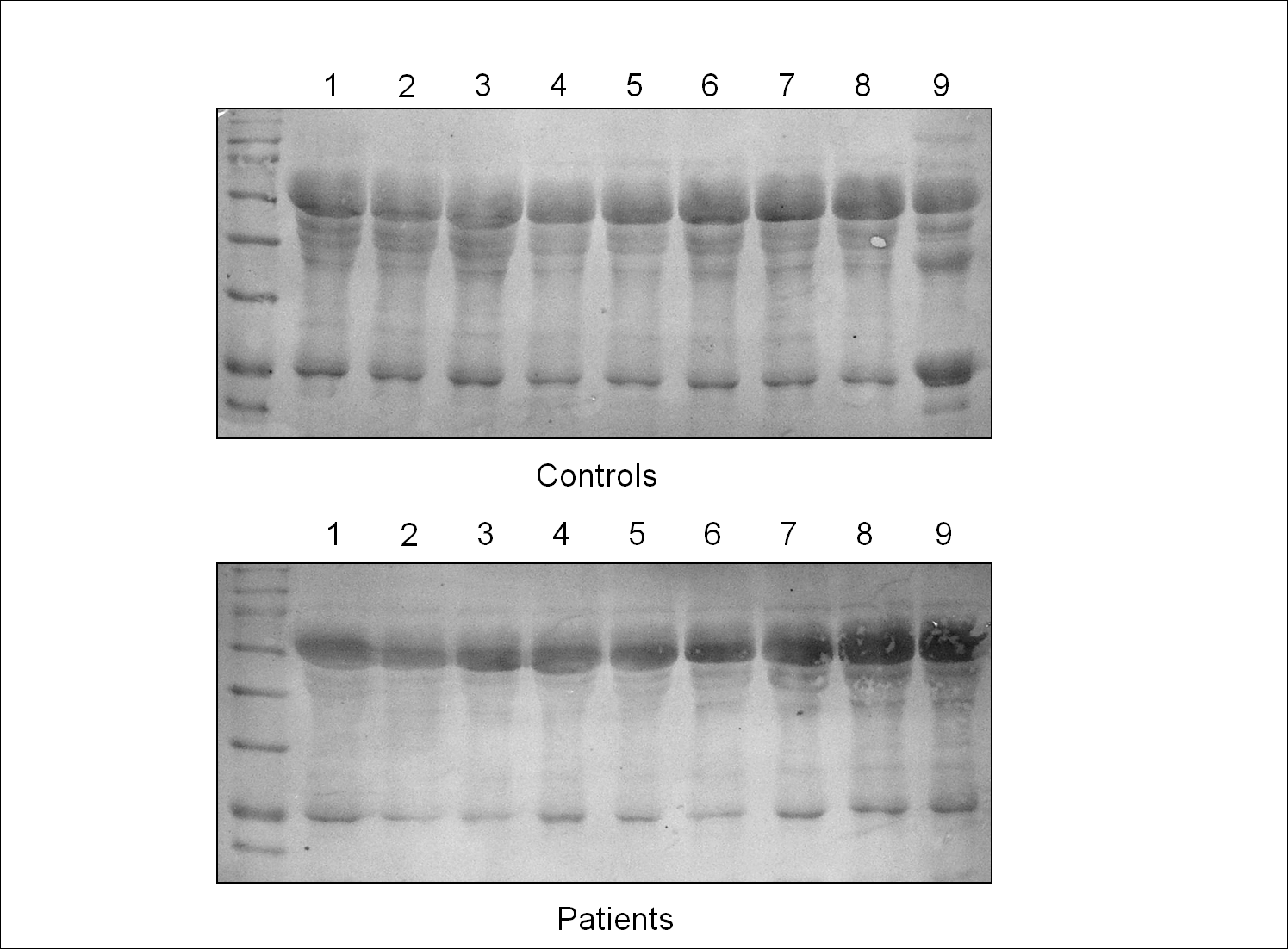

Supplement: Figure S2 — Ponceau image for densitometric analysis. Ponceau stained image of individual controls and RA patients after transferring the proteins to nitrocellulose membrane. (TIF) [file pone.0046374.s002.tif]
