# Supplementary material for: Jacalin Bound Plasma O-Glycoproteome and Reduced Sialylation of Alpha 2-HS Glycoprotein (A2HSG) in Rheumatoid Arthritis Patients
Source: PLoS One. 2012 Oct 3;7(10):e46374. doi: 10.1371/journal.pone.0046374 (PMC3463590; doi:10.1371/journal.pone.0046374)
Supplement: Table S1 — Demographic data of RA patients. (DOC) [file pone.0046374.s003.doc]

**Table 1S**: Description of RA patients used in the study.

|  | Male/ female | Age | CRP | RF | ESR | Disease duration |
| --- | --- | --- | --- | --- | --- | --- |
| Group 1 | 7/8 (n=15) | 35.8 ± 3.8 | 43.53 ± 34.4 | 218.8 ± 63.9 | 47.14 ± 25.0 | 4.56 ± 2.7 |
| Group 2 | 10/10 (n=20) | 46.7 ± 3.34 | 47.3 ± 22.7 | 338.66 ± 123.19 | 27.11 ± 14.67 | 5.87 ± 2.9 |

Values are presented as average ± S.D.
